# Probing the Energetic Metabolism of Resting Cysts under Different Conditions from Molecular and Physiological Perspectives in the Harmful Algal Blooms-Forming Dinoflagellate *Scrippsiella trochoidea*

**DOI:** 10.3390/ijms22147325

**Published:** 2021-07-07

**Authors:** Fengting Li, Aoao Yang, Zhangxi Hu, Siheng Lin, Yunyan Deng, Ying Zhong Tang

**Affiliations:** 1 CAS Key Laboratory of Marine Ecology and Environmental Sciences, Institute of Oceanology, Chinese Academy of Sciences, Qingdao 266071, China; lifengting@qdio.ac.cn (F.L.); yangaoao@lyu.edu.cn (A.Y.); zhu@qdio.ac.cn (Z.H.); lsh2246@mnnu.edu.cn (S.L.); 2 University of Chinese Academy of Sciences, Beijing 100049, China; 3 Laboratory for Marine Ecology and Environmental Science, Qingdao National Laboratory for Marine Science and Technology, Qingdao 266237, China; 4 Center for Ocean Mega-Science, Chinese Academy of Sciences, Qingdao 266071, China

**Keywords:** ATP content, ATP synthase subunit beta (*β-F_1_-ATPase*), energetic metabolism, resting cyst, *Scrippsiella trochoidea*, suppression subtractive hybridization (SSH), viability

## Abstract

Energetic metabolism is essential in maintaining the viability of all organisms. Resting cysts play important roles in the ecology of dinoflagellates, particularly for harmful algal blooms (HABs)-causative species. However, the energetic metabolism underlying the germination potency maintenance of resting cysts of dinoflagellate have been extremely scarce in studies from physiological and, particularly, molecular perspectives. Therefore, we used the cosmopolitan *Scrippsiella trochoidea* as a representative of HABs-forming and cyst-producing dinoflagellates in this work to obtain novel insights into the molecular mechanisms, regulating the energetic metabolism in dinoflagellate resting cysts, under different physical condition. As the starting step, we established a cDNA subtractive library via suppression subtractive hybridization (SSH) technology, from which we screened an incomplete sequence for the *β* subunit of ATP synthase gene (*β-F_1_-ATPase*), a key indicator for the status of cell’s energetic metabolism. The full-length cDNA of *β-F_1_-ATPase* gene from *S.trochoidea* (*Stβ-F_1_-ATPase*) was then obtained via rapid amplification of cDNA ends (RACE) (Accession: MZ343333). Our real-time qPCR detections, in vegetative cells and resting cysts treated with different physical conditions, revealed that (1) the expression of *Stβ-F_1_-ATPase* in resting cysts was generally much lower than that in vegetative cells, and (2) the *Stβ-F_1_-ATPase* expressions in the resting cysts under darkness, lowered temperature, and anoxia, and during an extended duration of dormancy, were significantly lower than that in cysts under the condition normally used for culture-maintaining (a 12 h light:12 h dark cycle, 21 °C, aerobic, and newly harvested). Our detections of the viability (via Neutral Red staining) and cellular ATP content of resting cysts, at the conditions corresponding to the abovementioned treatments, showed that both the viability and ATP content decreased rapidly within 12 h and then maintained at low levels within the 4-day experimentation under all the three conditions applied (4 °C, darkness, and anoxia), which are well in accordance with the measurements of the transcription of *Stβ-F_1_-ATPase*. These results demonstrated that the energy consumption of resting cysts reaches a low, but somehow stable, level within a short time period and is lower at low temperature, darkness, and anoxia than that at ambient temperature. Our work provides an important basis for explaining that resting cysts survive long-term darkness and low temperature in marine sediments from molecular and physiological levels.

## 1. Introduction

Harmful algae blooms (HABs) are ecological phenomenon that pose serious impacts on ecosystems, economy, and public health and have been increasing globally [1]. Dinoflagellates receive particular attention because approximately 75% of HAB events were caused by species of this group of microalgae [2]. As great competitors for ecological dominance under certain circumstances, their success, to some extent, stems from a suite of ecological strategies, such as toxin production, allelopathic effect, and nutritional flexibility [1,3,4,5]. Moreover, several dinoflagellate species have a characteristic strategy in their life history, including a dormant stage, called resting cyst [6,7]. The encystment (i.e., formation of resting cysts) for most, if not all, species of dinoflagellates is associated with a sexual process whereby the fusion of two motile gametes yields a planozygote that can eventually develop into a non-motile resting cyst and then sink into sediments [8,9]. Such cysts have been shown to have essential functions in the biology and ecology of dinoflagellates, especially for HABs-causative species, since they are related with recombination of genes, generation and termination of blooms, expansion of biogeographical distribution, and self-protection from viruses, grazers, or parasite attacks [6,7,8,9,10,11,12,13,14,15,16,17]. Although resting cyst production was described in many species and considered for being a key adaptation or survival strategy of dinoflagellates, relatively little information had been obtained about the physiology of dormancy in resting cysts, particularly about the energetic metabolism. 

Resting cysts must rely on energetic metabolism to maintain cell survival during dormancy. They not only use energetic metabolism to complete the basic life process to maintain activity, but also to resist adverse environments, including darkness, lowered temperature, and anoxia. Previously, several “phenomenological” studies were conducted on the energetic metabolism mechanism of dinoflagellates. They have found that the formation of resting cysts was accompanied by the increase in energy storage substances and the decrease in metabolic activities [18,19,20,21,22]. Under darkness conditions, generally speaking, the chloroplast of mature resting cysts will shrink and lose its photosynthetic capacity [18], which may lead to the cells unable to synthesize energy. Kang et al. (2017), who describe a significant metabolism and respiration reduction in resting cysts, and a sterol-enriched red accumulation body in resting cysts, which was supposed to be an energy source during dormancy [22]. Molecular investigations on the energetic metabolism of resting cysts of dinoflagellates have been rare in the literature except for the pioneering works by Deng et al. (2017) [23] on resting cysts of *S. trochoidea.* and by Roy et al. (2014), on temporary cysts of *Lingulodinium polyedrum* [24]. A series of 45 differential expressed genes were identified to be relevant to energy metabolism basing on comparative analyses in resting cysts and vegetative cells of *S. trochoidea*, which include categories of glycolysis, citrate cycle (TCA cycle), glyoxylate pathway, and fatty acid metabolism [23]. Significantly lower oxygen consumption was detected in resting cysts than that in vegetative cells [23]. Roy et al. (2014) found that the intracellular energy metabolism was reduced highly, due to the change of the phosphorome [24], and there were significant differences in the levels of the phosphorome involved in nucleic acid metabolism between the vegetative cells and the cysts [24]. However, the endogenous regulatory factors and physiological mechanisms, that allow resting cysts to survive the dormant phase smoothly for a long time (even a century) [25], including the genes and their expressions underlying these biological processes, are still poorly known. In this paper, we did some studies to understand the changes of intracellular energetic metabolism in cysts during dormancy.

Adenosine triphosphate (ATP) is produced through a variety of pathways, including mitochondrial oxidative phosphorylation, in the process of cellular energetic metabolism, and it is the most direct energy source for organisms to perform cellular functions. The ATP synthase (ATPase) plays a central role in cellular energy conversion as it produces the ATP [26]. The ATPase, also called F_0_F_1_-ATP synthase, consists of two structural domains: the catalytic F_1_ domain and the membrane proton channel F_0_ domain [27,28,29,30,31]. It is a large multi-subunit complex built of at least 16 different subunits, among them is the subunit beta (β-F_1_-ATPase), one of the major subunits of catalytic F_1_ domain, which is the only site with catalytic activity in the structure of ATPase [28,30,31,32,33]. There have been many studies applying β-F_1_-ATPase to characterize the catalytic activity of ATPase [34,35,36]. Therefore, investigating β-F_1_-ATPase in resting cysts is helpful to understand the energetic metabolism mechanism during dormancy at the molecular level. 

In this study, the cosmopolitan, HABs-causative, and resting cyst-producing species, *Scrippsiella trochoidea,* was adopted as the representative species of dinoflagellates [23,37] to survey the differentially expressed genes, relevant to energetic metabolism, by applying the suppression subtractive hybridization (SSH) approach into resting cysts and vegetative cells, and then, to study the transcription pattern of the ATP synthase subunit beta gene (*Stβ-F_1_-ATPase*). Based on the selected sequences in the subtractive libraries, we obtained the full-length cDNA sequences of a *Stβ-F_1_-ATPase*, via rapid amplification of cDNA ends (RACE)-PCR, and then quantified its transcriptions in vegetative cells and resting cysts at different conditions by qPCR. In addition, staining assays on the resting cysts with a vital stain and measurements of the intracellular ATP content, using an ATP detector, were also performed in the context of examining the energetic metabolism of resting cysts. Our results provide clues for further exploitations on the molecular mechanisms underlying energetic metabolism during dormancy maintenance of dinoflagellates.

## 2. Results

### 2.1. Gene Set Obtained from SSH Libraries

**Figure 1:**
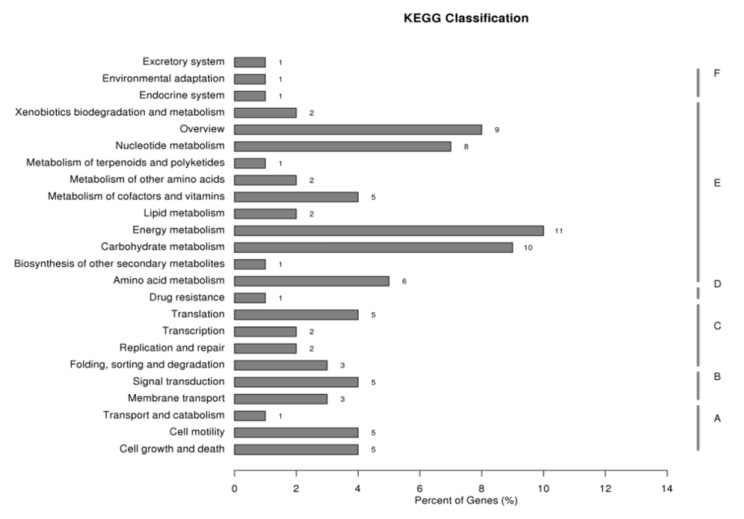
KEGG classification figure. KEGG (Kyoto Encyclopedia of Genes and Genome) is a database for genome deciphering, which is often used to predict the role of protein interaction networks in a variety of cellular activities. In this figure, A stands for cellular processed; B stands for environmental information processing; C stands for genetic information processing; D stands for human diseases; E stands for metabolism; F stands for organismal systems. We used *p* < 0.05 as a standard to find out the significant enrichment of pathways in DEGs.

The cDNA subtractive libraries of the vegetative cells and resting cysts of *S.trochoidea* successfully constructed using SSH approach contained 925 sequences with lengths greater than 100 bp, ranging from 102 to 1806 bp. After splicing, 280 sequences were obtained, 74.3% of which were annotated in at least one of the 6 public databases, non-redundant protein database (NR), eukaryotic Ortholog Groups (KOG), Kyoto Encyclopedia of Genes and Genomes (KEGG), KEGG Ortholog (KO), Gene Ontology (GO), and SWISS-PROT (Table 1). Among the contigs/sequences with GO annotations, 187 of which were annotated to be involved in biological processes, of which 55 were involved in metabolic processes, while 142 were annotated to be associated with molecular functions (Table 2). In the KEGG annotations, 57 sequences were annotated into metabolic pathways, accounting for 70% of all sequences that could be annotated in the KEGG database, and 20% of these 57 sequences could be annotated into the energy metabolism (Figure 1). Due to the central role played by ATPase in the energetic metabolism, a 159 bp fragment annotated as gene encoding “*β* subunit of ATP synthase” was selected for further characterizations (see below). 

### 2.2. Characterization of the Full-Length cDNA Sequence of Stβ-F_1_-ATPase

**Figure 2:**
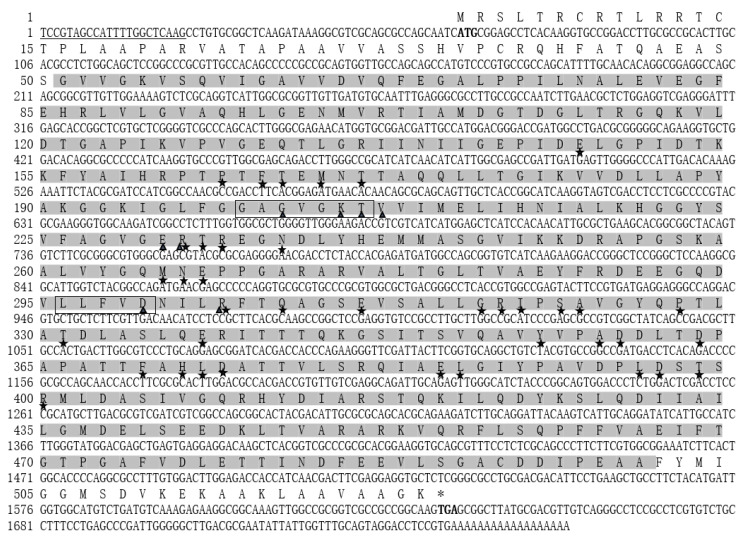
The full-length cDNA sequence and deduced amino acid sequence of *Stβ-F_1_-ATPase* (Accession: MZ343333). Sequence analysis was performed using NCBI database and numbered on the left. The start and stop codon are in bold; the conserved dinoflagellate spliced leader (DinoSL) is underlined in the 5′-UTR; the conserved domain is shaded in dark gray; the ATP binding sites are highlighted with triangles; the walker A/B motifs are boxed; the alpha submit interaction interfaces are highlighted with pentagram.

**Figure 3:**
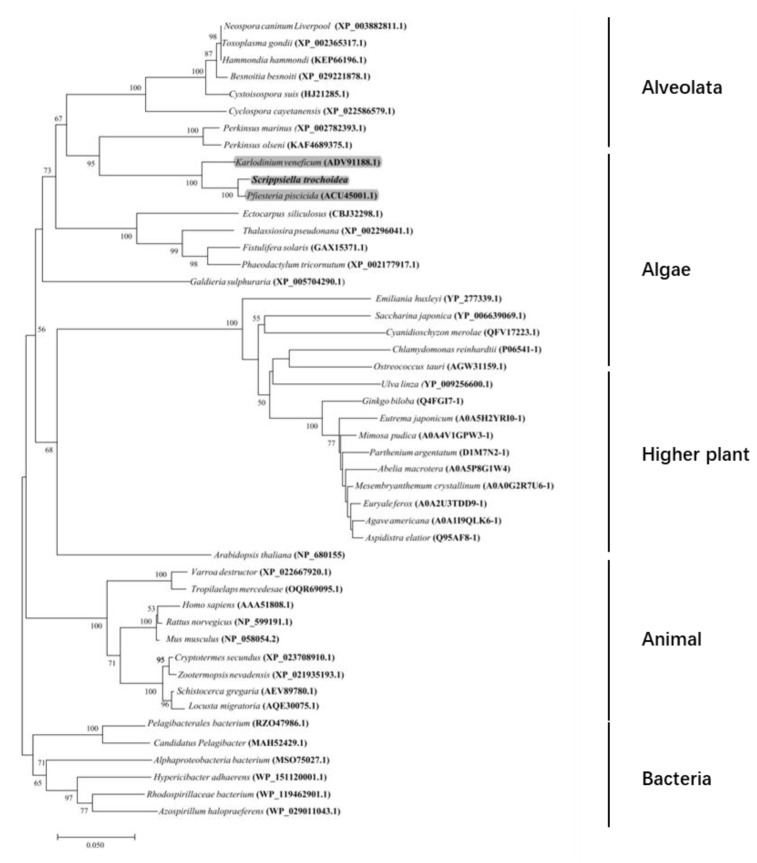
Phylogenetic tree inferred from *Stβ-F_1_-ATPase* amino acid sequences alignment using the neighbor-joining (NJ) method and its posterior probability shown. The dinoflagellate species are shown in gray. The new sequence is shown in bold.

The full-length of *Stβ-F_1_-ATPase* was obtained to be 1760 bp by overlapping the 3′ and 5′ RACE products (853 bp and 748 bp, respectively). The obtained sequence was deposited in GeneBank with accession number MZ343333. The *Stβ-F_1_-ATPase* contained a 63 bp-long untranslated region (UTR) at the 5′ end, a 122 bp UTR at the 3′ end, and a 1575 bp ORF, which encoded a protein of 524 amino acid residues with a predicted molecular weight of 55.95 kDa and a theoretical isoelectric point of 5.58. Analysis of the conserved domain in the deduced amino acid sequence of *Stβ-F_1_-ATPase* identified some specific hits, including ATP binding sites (Gly22; Lys9; Thr19; Val22; Glu12; Arg13; Asp12; Arg19), walker A/B motifs (GAGVGKT and LLFVD), and alpha submit interaction interfaces (Glu9; Pro12; Phe5; Thr15; Met4; Thr16; Arg14; Thr20; Arg15; Asn16; Met8; Asn17; Glu15; Arg19; Gln12; Glu19; Gly36; Arg20; Ile19; Ser12; Ala35; Pro18; Thr22; Glu20; Tyr8; Ala39; Asp16; Phe11; His9; Leu34; Asp17; Glu21; Leu36; Leu37; Asp19; Thr35; Arg24) (Figure 2). The *Stβ-F_1_-ATPase* protein shared 72–98% identities with other 47 species, including *Besnoitia besnoiti* (XP_029221878.1), *Hammondia hammondi* (KEP66196.1), *Perkinsus marinus* (XP_002782393.1), *Perkinsus olseni* (KAF4689375.1), *Neospora caninum Liverpool* (XsssP_003882811.1), *Cystoisospora suis* (PHJ21285.1), *Toxoplasma gondii MAS* (KFH15380.1), *Cyclospora cayetanensis* (XP_022586579.1), *Karlodinium veneficum* (ADV91188.1), *Pfiesteria piscicida* (ACU45001.1), *Saccharina japonica* (YP_006639069.1), *Ulva linza* (YP_009256600.1), *Galdieria sulphuraria* (XP_005704290.1), *Fistulifera solaris* (GAX15371.1), *Phaeodactylum tricornutum* (XP_002177917.1), *Thalassiosira pseudonana* (XP_002296041.1), *Ectocarpus siliculosus* (CBJ32298.1), *Chlamydomonas reinhardtii* (NP_958414.1), *Ostreococcus tauri* (AGW31159.1), *Cyanidioschyzon merolae* (QFV17223.1), *Emiliania huxleyi* (YP_277339.1), *Varroa destructor* (XP_022667920.1), *Cryptotermes secundus* (XP_023708910.1), *Schistocerca gregaria* (AEV89780.1), *Tropilaelaps mercedesae* (OQR69095.1), Zootermopsis nevadensis (XP_021935193.1), *Locusta migratoria* (AQE30075.1), *Homo sapiens* (AAA51808.1), *Mus musculus* (NP_058054.2), *Rattus norvegicus* (NP_599191.1), *Alphaproteobacteria bacterium* (MSO75027.1), *Rhodospirillaceae bacterium* (WP_119462901.1), *Pelagibacterales bacterium* (RZO47986.1), *Candidatus Pelagibacter* (MAH52429.1), *Hypericibacter adhaerens* (WP_151120001.1), *Azospirillum halopraeferens* (WP_029011043.1), *Abelia macrotera* (A0A5P8G1W4), *Ginkgo biloba* (Q4FGI7-1), *Arabidopsis thaliana* (NP_680155), *Agave americana* (A0A1I9QLK6-1), *Mimosa pudica* (A0A4V1GPW3- 1), *Aspidistra elatior* (Q95AF8-1), *Euryale ferox* (A0A2U3TDD9-1), *Eutrema japonicum* (A0A5H2YRI0-1), *Parthenium argentatum* (D1M7N2-1), and *Mesembryanthemum crystallinum* (A0A0G2R7U6-1) (see Supplementary Materials 2 for more details). Our neighbor-joining (NJ) tree also showed that the new yielded *Stβ-F_1_-ATPase* was clustered together with the dinoflagellate species, *Pfiesteria piscicida* and *Karlodinium veneficum*, closed to alveolata, and formed sister branches with algae (Figure 3).

### 2.3. Transcriptional Profiles of Stβ-F_1_-ATPase

**Figure 4:**
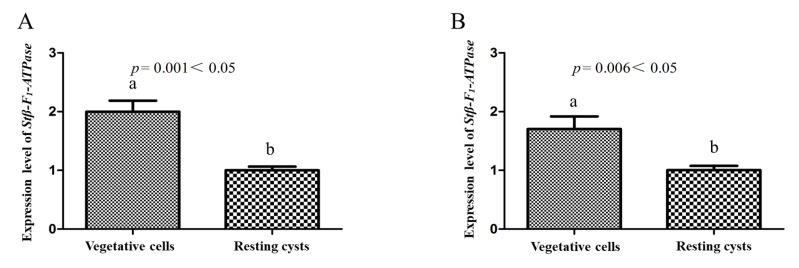
*Stβ-F_1_-ATPase* transcription levels relative to *CYC* (**A**) and *PEPCK* (**B**) in vegetative cells and resting cysts. Data were presented in graphs as mean ± standard deviation (SD), subjected to one-way analysis of variance (ANOVA) and a subsequent Tukey’s honestly significant difference test, *n* = 3, *p* < 0.05. “a” and “b” mean that there are differences between the groups they represent respectively.

**Figure 5:**
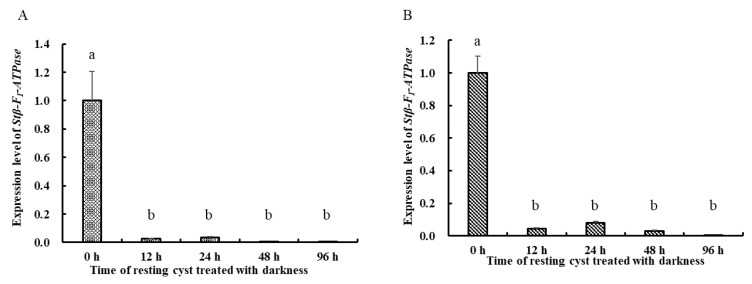
*Stβ-F_1_-ATPase* transcription levels relative to *CYC* (**A**) and *PEPCK* (**B**) when resting cysts treated with darkness. The expression level to % change relative to *CYC* and *PEPCK*. Data were presented in graphs as mean ± standard deviation (SD), subjected to one-way analysis of variance (ANOVA) and a subsequent Tukey’s honestly significant difference test, *n* = 3, *p* < 0.05. “a” and “b” mean that there are differences between the groups they represent respectively.

**Figure 6:**
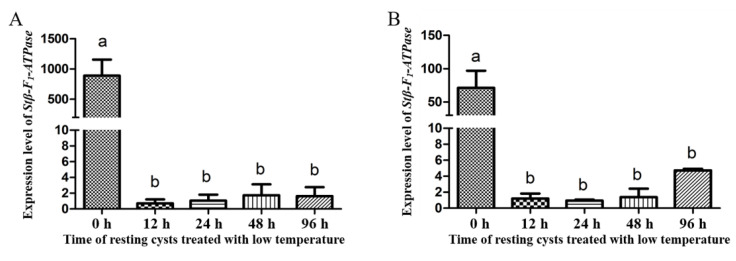
*Stβ-F_1_-ATPase* transcription levels relative to *CYC* (**A**) and *PEPCK* (**B**) when resting cysts treated with low temperature. Data were presented in graphs as mean ± standard deviation (SD), subjected to one-way analysis of variance (ANOVA) and a subsequent Tukey’s honestly significant difference test, *n* = 3, *p* < 0.05. “a” and “b”mean that there are differences between the groups they represent respectively.

**Figure 7:**
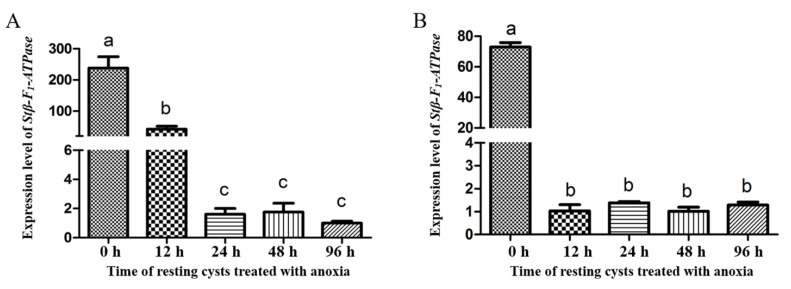
*Stβ-F_1_-ATPase* transcription levels relative to *CYC* (**A**) and *PEPCK* (**B**) when resting cyst treated with anaerobic conditions. Data were presented in graphs as mean ± standard deviation (SD), subjected to one-way analysis of variance (ANOVA) and a subsequent Tukey’s honestly significant difference test, *n* = 3, *p* < 0.05. “a”, “b” and “c” mean that there are differences between the groups they represent respectively.

Regarding the expression levels of *Stβ-F*_1_*-ATPase* at different life stages, *Stβ-F*_1_*-ATPase* expression in vegetative cells was significantly higher than that in newly harvested resting cysts, as reflected both in the results normalized to reference genes of **CYC** and **PEPCK** (Figure 4A,B). Under darkness, the expression of *Stβ-F*_1_*-ATPase* in resting cysts decreased compared with 0 h (Figure 5). In addition, there was a slightly increased expression at 24 h and a dramatic decrease (i.e., 0.14% of the initial expression. The transcriptional level at 0 h was too high, the statistical analysis showed that there were significant differences between the 0 h and the other groups, while there was no significant difference among the other groups). Regarding the expression of *Stβ-F*_1_*-ATPase* under lowered temperature, it was obviously lower than that at 0 h and then it decreased rapidly to a very low level within 12 h, with no significant difference during the following 84 h (Figure 6). The expression of *Stβ-F*_1_*-ATPase* in resting cysts, under anoxia conditions, was observed to decrease rapidly within 12 h (*CYC* normalized) or 24 h (*PEPCK* normalized) and then reached a relatively low and stable level at 96 h (Figure 7).

### 2.4. NR Staining-Defined Viability of Resting Cysts during Dormancy

**Figure 8:**
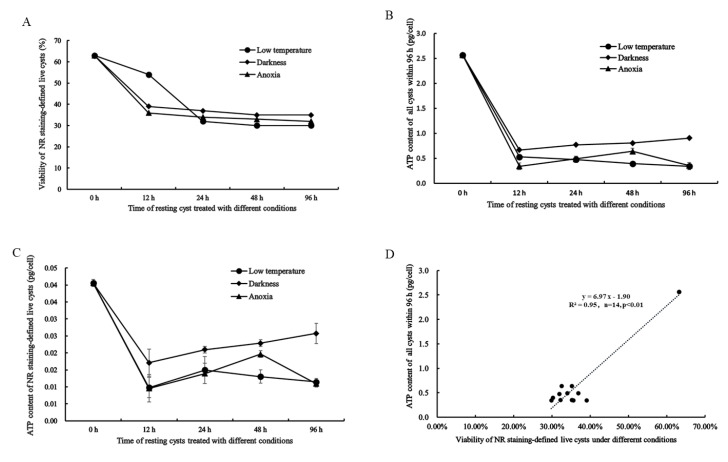
The activity of energetic metabolism in resting cysts as reflected in viability and ATP content measured at lowered temperature (4 °C), darkness and anoxia. (**A**) The viability of resting cysts measured with NR-staining. (**B**) Cellular ATP content of all cysts in sample. (**C**) Cellular ATP content of NR staining-defined live cysts. (**D**) Regression analysis between the cellular ATP content and the NR staining-defined viability, R^2^ = 0.95, *n* = 14, *p* < 0.001.

The viability of resting cysts measured with NR-staining under darkness, low temperature, and anoxic conditions was observed to decrease drastically (from 63.21 to 29.82%) within 24 h for all three conditions and then keep at a relatively stable level (30–35%; Figure 8A). At 12 h, the viability of cysts at 4 °C was higher than that at darkness and anoxia, but after 24 h, the viability of cysts under all conditions reached a similarly low level (Figure 8A).

### 2.5. Cellular ATP Content in All and Live Resting Cysts and Its Relation to NR Staining-Defined Viability

It should be noted, first, that the cellular ATP content was measured for both all cysts in a sample (Figure 8B) and live cysts that were determined using NR staining as described above (Figure 8C). With the extended duration of dormancy (0–96 h) for the cysts at the three conditions (darkness, 4 °C, and anoxia), the cellular content of ATP for both all cysts and live cysts generally decreased significantly within the first 12 h and remained steadily low between 12–96 h (Figure 8B,C). However, between 12–96 h, the cellular ATP contents calculated for live cysts exhibited more fluctuations (e.g., a slight increase for the cysts in darkness) than was calculated for all cysts (Figure 8B vs. Figure 8C). Regression analysis demonstrated a significant positive correlation between the cellular ATP content for all cysts at the three conditions and the NR staining-defined viability (R^2^ = 0.95, n = 14, *p* < 0.001; Figure 8D), although there was an outlier and the data dots were not evenly distributed along the fitting curve (linear) due to the drastic decrease both in the viability and cellular ATP content (Figure 8D).

## 3. Discussion

### 3.1. Comments on the Results and Limitations of SSH

SSH had been a widely used approach to probe species-specific functional genes, prior to the widespread application of the high-throughput next generation sequencing (NGS), and has contributed, substantially, to many investigations [38,39,40,41,42]. According to Akopyants and Fradkov (1998), PCR-based subtractive hybridization method could effectively detect the special gene of a certain strain and apply it to the gastric pathogen *Helicobacter pylori* [38]; Lamar and Palmer (1984) have investigated the structure of the murine Y chromosome by first developing a novel method for specifically cloning Y-encoded DNA and then generating a library enriched for Y-specific DNA sequences [42]. Our SSH screening totally obtained 280 contigs and 74.3% of them could be annotated in at least one of the databases. From the sequences annotated to genes relevant to energetic metabolism, we confirmed *Stβ-F_1_-ATPase* gene in *S. trochoidea*. The resting contigs (25.7% of 280) were not annotated to any record of those databases, which might be partly due to these sequences being too short. It is also possible that these sequences are only present in some dinoflagellate species which have not been included in the databases. Due to the extremely large genome sizes of dinoflagellates [43,44] and too few genomes of dinoflagellate species having been fully sequenced, it is not surprising to have many genes from *S. trochoidea* that are not able to be annotated to a particular function. Our previous investigation on the transcriptome of resting cysts in *S. trochoidea* has obtained some important insights into the energetic metabolism mechanisms of resting cysts at the molecular level, including the differentially expressed genes (DEGs) relevant to the pathways of electron transport chain, carbon metabolism, amino acid metabolism, and many other pathways [23]. Compared to more than 3400 DEGs between resting cysts and vegetative cells of *S. trochoidea*, observed via the transcriptomic study [23], the number of 280 contigs, obtained via SSH, in this work is surely a partial gathering. Nevertheless, this preliminary screening provided us a start point from which we selected the particular gene that plays vital roles in the energetic metabolism and thus dormancy of dinoflagellate cysts. 

### 3.2. The Activity of Energetic Metabolism in Resting Cysts as Reflected in the Expression of Stβ-F_1_-ATPase, Viability and ATP Measurements

Molecular investigations on the energetic metabolism of resting cysts of dinoflagellates have been rare in the literature except for the pioneering works by Deng et al. [23], on resting cysts of *S. trochoidea*, and by Roy et al. (2014) on temporary cysts of *L. polyedrum* [24]. The present work obtained the full-length cDNA of *Stβ-F_1_-ATPase*, having the complete coding region for 524 amino acid residues, similar in length to those of other dinoflagellates such as *Pfiesteria piscicida* (427 aa; ACU45001.1) and *Karlodinium veneficum* (514aa; ADV91188.1). Across 47 species from different taxa, *β-F_1_-ATPase* shows a high degree of homology, which is in accordance with previous notions that *β-F_1_-ATPase* appeared to be highly conserved within the lineage of an organism [28,31,45,46]. ATP synthase is a key enzyme in energy metabolism, which can directly synthesize ATP from ADP and Pi [29,31]. The *β* subunit is one of the most important structures of ATP synthase, which contains the catalytic site of the enzyme [29,30,47]. Any change in the expression of *β-F_1_-ATPase* in resting cysts, thus, indicates a corresponding change of the ATP synthesis and the activity of energetic metabolism. 

Here, we analyzed the differential expressions of *Stβ-F_1_-ATPase* in *S. trochoidea* at different stages of life cycle and different states of its resting cysts. In addition, we carried out physiological experiments to observe the parallel changes among the expression of *β-F_1_-ATPase*, the level of cellular ATP content, and viability of resting cysts under the dark, 4 °C, or anoxic conditions (i.e., physiological status of the cysts). Previous examinations confirmed that, compared with vegetative cells, resting cysts of dinoflagellates have a much reduced, or even negligible, level of cellular ATP and a reduced respiration level as well [18,19,20]. Interestingly, Roy et al. (2014) found that the overall metabolic level in the low temperature-induced temporary cysts of the dinoflagellate *L. polyedrum* was also significantly reduced in comparison to that of vegetative cells [24]. From Kang et al., [22], the reduced energy source of resting cysts during dormancy, it could be inferred that the expression of ATPase in resting cysts might be associated with consumption of the stored energy. More importantly, our recent transcriptomic study demonstrated significantly down-regulated expressions in genes relevant to energy metabolism (TCA cycle, glycolysis, and photosynthesis) in the resting cyst of *S. trochoidea* [23]. In terms of energetic metabolism, the observation of the current work that the expression level of *Stβ-F_1_-ATPase* in resting cysts was much lower than that in vegetative cells and that the gene expression changes with time under the three dormant conditions (darkness, lowered temperature, and anoxia) are all theoretically consistent to those studies described above. Considering the changes of cysts viability and cellular ATP contents together with the changing trend in the expression of *Stβ-F_1_-ATPase* under different dormant conditions, we concluded that, as the dormancy of resting cysts proceeds, the energy consumption in resting cysts drastically decreases to a minimal level within a short period. We speculated that, under the three extreme conditions (darkness, low temperature, and anoxia), temperature and oxygen level have the greatest impact on the cyst viability. However, we also speculate that many, if not all, cysts that were detected to be ‘dead’ by NR staining might be the cysts of which the physiological activity have reached a level, that the viability-indicative molecules could not be ‘visualized’ via NR staining [48,49,50]. While, admittedly, this needs further verification with more accurate measures for the viability detection of individual cells, our results for the gene expression of *Stβ-F_1_-ATPase*, viability measurement, and cellular ATP measurement are well consistent with each other. It is also worth mentioning that the expression level fluctuates, to a certain extent, within 24 h (Figure 5B in revision particularly), indicating that the resting cysts have an energy-consuming adjustment during the initial stage entering each treatment condition.

### 3.3. Further Discussion and Conclusions

The sexual reproduction and dormancy process of resting cysts are highly similar to, or analogous to, the seeds of higher plants. During the formation and germination of higher plant seeds, the expression of energy storage substances and genes related to energetic metabolism would change significantly [51]. Under low temperature conditions, the enzymatic reaction factors in cells that will be inhibited or even inactivated, thereby affecting the corresponding chemical process [52,53]. In a normal aerobic environment, cells mainly synthesize ATP efficiently through the tricarboxylic acid cycle (TCA) and other pathways, releasing a great deal of energy [54,55]. However, under anaerobic conditions, the TCA pathway cannot carry out normally and efficiently, so the ATP content in the cell may decrease. For the resting cysts in sediment, on one hand, they cannot obtain enough light for carbon fixation, and, on the other hand, they must rely on limited energy to maintain cell viability and retain sufficient energy for germination. Therefore, cysts during dormancy have to maintain a minimum energy consumption. This study observed that the levels of *Stβ-F_1_-ATPase* expression, NR staining-defined viability, and cellular ATP content in resting cyst drastically dropped to a very low level within 24 h under the three conditions, supposedly being optimal, for maintaining dormancy. These results should explain, to a large extent, why resting cysts of dinoflagellates can survive for more than 100 years [25,56] under darkness, lowered temperature, and anoxia in the field.

After all, although energetic metabolism is of vital importance to dinoflagellate resting cysts, our knowledge about the molecular mechanisms (e.g., the regulating genes involved and their interactions) of resting cyst formation, and germination, is still at the stage of infancy, and more intensive investigations are undoubtedly desirable.

## 4. Materials and Methods

### 4.1. Algal Cultures and Resting Cysts Harvesting

*S. trochoidea* (strain STBDH1) used in this study was established from Beidaihe, Hebei, China in August 2014 via single-cyst germination. All the microalgal cultures were cultured in sterilized natural seawater with f/2 (-Si) medium [57] (see Supplementary Materials 3 for more details) at the salinity of 32–33, and maintained at 20 ± 1 °C in an incubator (Ningbo Jiangnan Instrument Factory, Ningbo, China) with 12:12 h light:dark cycle and 100 μmol photons m^−2^∙s^−1^. Before inoculation, a penicillin-streptomycin solution (a mixture of 10,000 I.U. penicillin and 10,000 μg∙mL^−1^ streptomycin, Solarbio, Beijing, China) was added to the medium with a final concentration of 2% to inhibit bacterial growth. For transfers, cultures at exponential stage were inoculated into 500-mL flasks containing 300 mL medium to reach an initial cell density of ~1 × 10^3^ cells·mL^−1^. Resting cysts were produced by adding fine sands, as described in Yang et al., [58] and harvested from cultures at the room temperature (±21°). The collected resting cysts were then rinsed with appropriate amount of aseptic seawater (containing 0.05% Tween-80 and 0.01M EDTA) for 30 min, lysozyme (0.5 mg·mL^−1^) for 10 min, and SDS (0.25%) for 10 min at room temperature, and finally rinsed with aseptic seawater several times to remove the used reagent [59].

### 4.2. Treatments of Resting Cysts: Darkness, Lowered Temperature, and Anoxia

The darkness treatments were conducted via wrapping the cyst-containing tubes with aluminum foils and placing in the incubator with light and oxygen for 0, 12, 24, 48, and 96 h before putting samples into liquid nitrogen. The lowered-temperature treatments were conducted by placing cyst samples (tubes) at 4 °C with irradiation for 0, 12, 24, 48, and 96 h before terminating the experiment with liquid nitrogen. The anoxia treatments were conducted by fulfilling the cyst-containing centrifuge tubes (1.5 mL) with f/2 medium, placing in 21 °C incubator for 0, 12, 24, 48, and 96 h prior to being put in the liquid nitrogen. In the anoxia treatment group, the dissolved oxygen (DO) level of samples was monitored by a multiple water quality analyzer (Seven Excellence, Mettler Toledo, Switzerland) after treated with 0 h and 12 h, and the results were 8.9 mg∙L^−1^ and 0–0.01 mg∙L^−1^, respectively. This indicates that the method of our anoxia treatment can achieve the desired treatment effect. It is worthy of mentioning that all tubes treated with darkness, low temperature, and anoxia were filled with f/2 medium to minimize the effect of water evaporation and salinity change.

### 4.3. SSH Library Construction and Reverse Northern Blot Hybridization

Total RNA was extracted by RNeasy^®^ Plant Mini Kit (QIAGEN, Dutch Finn los, Germany). A SMARTer PCR cDNA Synthesis Kit (Clontech, Takara Biotechnology, Dalian, China) was used to reverse transcribe mRNA to cDNA, according to the manufacturer’s protocol. A cDNA subtractive library (resting cysts RNA as tester, vegetative cells RNA as driver) of resting cysts and vegetative cells was prepared according to a PCR-Select cDNA subtraction kit (Clontech). The cDNAs of tester and driver were digested with the enzyme *Rsa I* and linked to different adaptors, followed by two rounds of hybridizations and PCR-selected. The PCR products were cloned into the vector *pMD*-18T (Takara, Dalian, China) and transformed into *Escherichia coli* line. After blue-white spot screening and agarose gel electrophoresis, the PCR products of the screened positive clones were used to dot hybridization experiment to further screen the clones with significant differences. According to the protocol of DIG high prime DNA labeling and detection starter kit I (Roche, Basel, Switzerland), two membranes were made at the same time, and all the experimental treatments were the same except that the probes on the membranes (one was digoxigenin-labeled cDNA of resting cysts, the other was digoxigenin-labeled cDNA of vegetative cells) were different. Finally, the selected clones with significant differences were Sequenced (Sangon Biotech, Qingdao, China).

### 4.4. Sequencing and Alignment Analysis

All the obtained sequences were used for BLAST (BLASTX) search and annotation against public databases, including NR, KOG, KEGG, KO, GO and SWISS-PROT with 10^−5^ E-value cutoff. Functional annotation by GO terms was analyzed using Blast2go program [60]. WEGO [61] was used to classify GO function.

### 4.5. Full-Length cDNA Cloning of Stβ-F_1_-ATPase

To isolate the full-length cDNA of *Stβ-F_1_-ATPase*, rapid amplification of cDNA ends (RACE) PCR was conducted as described previously [37]. The obtained partial sequence of *β-F_1_-ATPase* from SSH library was used to design gene specific primer sets (Table 3) for 5′ and 3′ RACE, respectively. The RACE cloning was performed by nested PCR, and the template for the second PCR was obtained by diluting the first PCR product tenfold. For the 5′ end, the forward primer DinoSL [37,62] paired with reverse primers β-F_1_-ATPase-5′-F and β-F_1_-ATPase-5′-R (Table 3) were used with touchdown PCR protocol [63], while the forward primers β-F_1_-ATPase-3′-F and β-F_1_-ATPase-3′-R (Table 3) coupled with reverse primer GeneRacer3 (Invitrogen, Karlsruhe, Germany) were conducted in the same way as for the 5′ RACE. After reactions, PCR products were detected by electrophoresis in 1% agarose gel, and targeted bands were purified by a DNA gel extraction kit (TaKaRa, Tokyo, Japan) and then ligated into the p*EASY*-T1 cloning vector (TransGen Biotech, Beijing, China). Finally, all samples sequenced by company (TsingKe Biotech, Beijing, China).

### 4.6. Characteristics Analyses of Stβ-F_1_-ATPase

The ORF Finder program [64] was used to search ORF (open reading frame) in the generated full-length nucleotide sequences of *Stβ-F_1_-ATPase* The ProtParam program on ExPASy (http://www.expasy.ch/tools/protparam.html (accessed on 5 June 2021)) was used to predict the theoretical molecular weight and isoelectric point properties of amino acids sequences [65]; The phylogenetic tree was constructed by the NJ method using MEGA 7.0 software, and the confidence of each branch is calculated by using Bootstrap to repeat 500 times.

### 4.7. Validation of Real-Time Quantitative PCR (qPCR) Reference Genes Applicable for S. trochoidea Strain STBDH1 at Different Life Stages

Based on the transcriptomic sequencing data of this species (GenBank Accession No. SRP058465; [23]), 15 housekeeping genes (HKGs), glyceraldehyde-3-phosphate dehydrogenase (*GAPDH*), actin (*ACT*), cytochrome oxidase subunit 1 (*COX1*), cyclophilin (*CYC*), phosphoenolpyruvate carboxylase-related kinase (*PEPCK*), ubiquitin conjugating enzyme (*UBC*), *α*-tubulin (*TUA*)*,* *β*-tubulin (*TUB*), S4 ribosomal protein (*Rp-S4*), S-adenosyl methionine synthetase (*SAM*), ubiquitin (*UBQ*), malate dehydrogenase (*MDH*), elongation factor G (*EF-G*), eukaryotic initiation factor 4E (*Eif4e*), and luciferin-binding protein (*LBP*), were selected to assess whether their expressions are stable enough as reference genes to study functional gene expression in *S. trochoidea* strain STBDH1 at different life stages. Gene expression stabilities were estimated with 3 softwares: geNorm [66], NormFinder [67], and BestKeeper [68,69,70] (see Supplementary Materials 1 for more details).

### 4.8. Transcriptional Profiles of Stβ-F_1_-ATPase with qPCR Detection

Based on the results of validation described above (Supplementary Material 1), the reference genes combination, *CYC* and *PEPCK*, was used in the subsequent qPCR detections of *Stβ-F_1_-ATPase* expression profile in *S. trochoidea* at different life cycle stages. The qPCRs were performed on Bio-Rad CFX96 Real-Time PCR Detection System using Takara Green Premix Ex Taq^TM^ II (Tokyo, Japan). The protocol and cycling conditions generally followed that described in Deng et al. [37]. The specific primer set, qβ-F_1_-ATPase-F and qβ-F_1_-ATPase-R (Table 3), was used to amplify 156 bp product of *Stβ-F_1_-ATPase*. The reactions were performed in biological triplicate with the following cycling conditions: 95 °C for 30 s; 95 °C for 10 s and 50 °C for 30 s, then 72 °C for 30 s (40 cycles). The melting curve confirmed the specificity of each pair of primers, and the relative standard curves verified the amplification efficiency of all primers [71,72]. According to the Ct value obtained by the instrument, the expression difference was calculated using 2^−^^△△Ct^ relative quantification method [73]. All the experimental data were subjected to one-way analysis of variance (ANOVA), and significance was inferred when *p* ≤ 0.05. SPASS 22 was used for statistical analysis.

### 4.9. Measurements for the Viability of S. trochoidea Cysts via Neutral Red (NR) Staining

Neutral red has been shown being a sound vital stain to evaluate viability of live cells or zooplankton [49,50,74,75,76] and can be conveniently observed under bright field light microscopy. Therefore, NR was adopted to assess the viability of resting cysts. The stock solution of NR (with a concentration of 0.5%) (Solarbio Science & Technology, Beijing, China) was diluted into 0.33% working solution and the pH was adjusted to around 7 in order to intensify the signal strength. After obtaining the resting cysts treated above, 1.0 mL per well of NR solution was gently mixed with cysts and stained at room temperature for 24 h (a prolonged staining time was adopted due to the generally low physiological activity of cysts). All treatments have triplicate samples, each containing ~1500 cells. Cells were observed under an inverted microscope (IX73, Olympus, Japan) using a Sedgewick Rafter counting chamber (1.0 mL of sample). The cysts stained red by NR were considered being viable, and the viability of each sample was calculated as the percent of ‘viable’ cysts in the total number of cysts examined (%).

### 4.10. Measurements of the Cellular ATP Content in Cysts

For all cyst samples those were treated as described above, their cellular ATP contents were also measured. Cysts were first crushed using a TGrinder High Speed Tissue grinder (OSE-Y30, Tiangen Biotech, Beijing, China) at room temperature for 90 s. The intracellular ATP content was quantified using QuenchGoneTM Aqueous Test Kit (LuminUltra, Canada; [77,78]) spectrophotometer (LuminUltra, Canada). After the RLU (relative light units) was obtained, the cellular ATP content was calculated using the formula: cATP = RLU_cATP_/RLU_ATP1_ ∗ 10,000/N_sample_, where cATP denotes cellular ATP content, RLU the relative light units which is Lux/OD600, and N_sample_ the number of live cysts, as described above.

## Figures and Tables

**Table 1:** The number and proportion of genes that successfully annotated in public databases.

Database	Number of Comments	Comment Percentage (%)
NR	204	72.86
KOG	40	14.29
KO	112	40
KEGG	58	20.71
GO	112	40
SWISS-PROT	108	38.57
All Databases	21	7.50
At least one Database	208	74.29
Unannotated	72	25.71
Total	280	100

**Table 2:** The sequence distribution of Gene Ontology (GO) function classification.

Classification of GO	GO-Id	Gene Function	Sequence Number	Percentage of GO-Annotated Genes	Percentage of Total Sequence
Cellular component	GO:0005623	cell	41	36.61	14.64
	GO:0044464	cell part	41	36.61	14.64
	GO:0032991	macromolecular complex	4	3.57	1.43
	GO:0043226	organelle	3	2.68	1.07
	GO:0031975	envelope	2	1.79	0.71
Molecular function	GO:0003824	catalytic activity	61	54.46	21.79
	GO:0005488	binding	40	35.71	14.29
	GO:0060089	molecular transducer activity	19	16.96	6.79
	GO:0005215	transporter activity	12	10.71	4.29
	GO:0005198	structural molecule activity	6	5.36	2.14
	GO:0030528	transcription regulator activity	2	1.79	0.71
	GO:0009055	electron carrier activity	2	1.79	0.71
	GO:0003824	catalytic activity	61	54.46	21.79
Biological process	GO:0008152	metabolic process	55	49.11	19.64

**Table 3:** List of primers used in the present study.

Primer Name	Primer Sequences (5′→3′)	Remarks
β-F_1_-ATPase-3′-F	GATGAGGAGGGCCAGGACGTGC	3′ RACE
β-F_1_-ATPase -3′-R	AGATGAACGAGCCCCCAGGTGC	3′ RACE
β-F_1_-ATPase -5′-F	ACCTGGGGGCTCGTTCATCTGGC	5′ RACE
β-F_1_-ATPase -5′-R	CTTGCGTGAAGCGGAGGATGTTG	5′ RACE
qβ-F_1_-ATPase-F	CCAACAGTGCCGATACCT	*Stβ-F_1_-ATPase* qPCR
qβ-F_1_-ATPase-R	GCCAGATGAACGAGCC	*Stβ-F_1_-ATPase* qPCR
CYC-F	CTACGAATGGTGGGAGACG	*CYC* qPCR
CYC-R	TCGCAAGTTAGCGGGACT	*CYC* qPCR
PEPCK-F	GAATGCCACCGTTGAGTTG	*PEPCK* qPCR
PEPCK-R	CTCCGCGAGTGAATGTGC	*PEPCK* qPCR

## Data Availability

The data is contained within the article and supplementary material.

## Supplementary Materials

The following are available online at https://www.mdpi.com/article/10.3390/ijms22147325/s1.
