# Supplementary material for: Probing the Energetic Metabolism of Resting Cysts under Different Conditions from Molecular and Physiological Perspectives in the Harmful Algal Blooms-Forming Dinoflagellate Scrippsiella trochoidea
Source: Int J Mol Sci. 2021 Jul 7;22(14):7325. doi: 10.3390/ijms22147325 (PMC8307125; doi:10.3390/ijms22147325)
Supplement: Supplementary file 1 [file ijms-22-07325-s001.zip › Supplementary Materials 1.pdf]

Supplementary materials 1. Probing the energetic metabolism of resting cysts under different conditions from molecular and physiological perspectives in the harmful algal blooms-forming dinoflagellate *Scrippsiella trochoidea*

1. Material and method

S. trochoidea (strain STBDH1) used in this study was established from Beidaihe, Hebei, China in August 2014 via single-cyst germination. All the microalgal cultures were cultured in sterilized natural seawater with f/2 (-Si) medium [1] at the salinity of 32-33, and maintained at 20±1℃ in an incubator (Ningbo Jiangnan Instrument Factory, China) with 12: 12 h light:dark cycle and 100 μmol photons $m^{-2}{\cdot}s^{-1}$. Before inoculation, a penicillin-streptomycin solution (a mixture of 10,000 I.U. penicillin and 10,000 $\mu g{\cdot}mL^{-1}$ streptomycin, Solarbio, Beijing, China) was added to the medium with a final concentration of 2% to inhibit bacterial growth. For transfers, cultures at exponential stage were inoculated into 500-mL flasks containing 300 mL medium to reach an initial cell density of ~ $1{\times}10^3$ $cells{\cdot}ml^{-1}$. Resting cysts were produced by adding fine sands as described in Yang et al., [2] and harvested from cultures at the room temperature. The collected resting cysts were then rinsed with appropriate amount of aseptic seawater (containing 0.05 % Tween-80 and 0.01M EDTA) for 30 min, lysozyme (0.5 $mg{\cdot}ml^{-1}$) for 10 min, and SDS (0.25 %) for 10 min at room temperature, and finally rinsed with aseptic seawater several times to remove the used reagent [3]. After collecting the cells, put them immediately in liquid nitrogen, and stored at -80°C before RNA extraction.

Based on the transcriptomic sequencing data of this species (GenBank Accession No. SRP058465; [4]), 15 housekeeping genes (HKGs), glyceraldehyde-3-phosphate dehydrogenase (*GAPDH*), actin (*ACT*), cytochrome oxidase subunit 1 (*COX1*), cyclophilin (*CYC*), phosphoenolpyruvate carboxylase-related kinase (*PEPCK*), ubiquitin conjugating enzyme (*UBC*), *α*-tubulin (*TUA*), *β*-tubulin (*TUB*), S4 ribosomal protein (*Rp-S4*), S-adenosyl methionine synthetase (*SAM*), ubiquitin (*UBQ*), malate dehydrogenase (*MDH*), elongation factor G (*EF-G*), eukaryotic initiation factor 4E (*Eif4e*) and luciferin-binding protein (*LBP*), were selected to assess whether their expressions are stable enough as reference genes to study functional genes expression in *S. trochoidea* strain STBDH1 at different life stages. Total RNA was extracted separately from vegetative cells and resting cysts by RNeasy® Plant Mini Kit (QIAGEN, Germany), and treated with an RNase-free DNase Set (QIAGEN) to eliminate the genomic DNA. The quantity and quality of total RNA were analyzed by Nonadrop 1000 (Thermo Fisher Scientific, USA). The synthesis of first strand cDNA was prepared with the PrimeScript II cDNA synthesis kit (TaKaRa, Japan) in according with the manufacturer's instructions (Table S1). The qPCRs were performed on Bio-Rad CFX96 Real-Time PCR Detection System using Takara Green Premix Ex Taq™ II (Tokyo, Japan). The reactions were performed in biological triplicate with the following cycling conditions: 95℃ for 30 s; 95℃ for 10 s and 50℃ for 30 s, then 72℃ for 30 s (40 cycles). The melting curve confirmed the specificity of each pair of primers, and the relative standard curves verified the amplification efficiency of all primers [5, 6]. According to the Ct value obtained by the instrument, the expression difference was calculated using $2^{-\triangle\triangle Ct}$ relative quantification method [7]. All the experimental data were subjected to one-way analysis of variance (ANOVA), and significance was inferred when $p \leq 0.05$. SPASS 22 was used for statistical analysis. Gene expression stabilities were estimated with 3 softwares: geNorm [8], NormFinder [9], and BestKeeper [10].

Table S1-1. List of primers used in the present study

Primer name	Sequences (5′→3′)
GAPDH-F	GTGGTGCCAAGAAGGTGATC
GAPDH-R	CAAGAGGCGTTCGAGACAA
TUA-F	AACACCTTCTTCAGCGAGACA
TUA-R	TTCCCAATGGTATAGTGACCC
TUB-F	CCAGTGCGGCAACCAGAT
TUB-R	CAGGCTCCAGGTCCATCA
ACT-F	CACGGCATTGTCACGAAC
ACT-R	CGTAGAGCGACAGAACAGC
COX1-F	TTGCCATGAGCTGTATTT
COX1-R	ATGACGGATTCCCAAGAT
Rp-S4-F	CCGCTCATCGTAATGCTCC
Rp-S4-R	CGCCTCCTCCTTCACTATCTT
CYC-F	CTACGAATGGTGGGAGACG
CYC-R	TCGCAAGTTAGCGGGACT
SAM-F	AGATTGCGTTTGATTGCC
SAM-R	TGATTCCCTGATGCGTGT
UBQ-F	AGCGACTACAACATCCAGAAGG
UBQ-R	AAGGCGGGCGTAGCACTT
MDH-F	GTTGGCTGCCTTGGACCCT
MDH-R	GCATCTGCGCTCGATGTG
EF-G-F	CTTCCCGACCTATTGTGG
EF-G-R	GAACGGCTCACTGGCATC
PEPCK-F	GAATGCCACCGTTGAGTTG
PEPCK-R	CTCCGCGAGTGAATGTGC
eIF4E-F	AGTGGGAGGACAAGATGAATG
eIF4E-R	GATCATGTTCGCTGGCTCA
LBP-F	ACAGACTTGGCTAACTATTGG
LBP-R	TGCTGAGCTGGACACGAT
UBC-F	GTCTTGACCTACTACGTGGAGC
UBC-R	CGGGCGTTGTACTGATGG

2. Result

According to the requirement that the Ct-values of 6 candidate reference genes were less than 30

and the dissolution curves were single peak, six genes including *GAPDH*, *ACT*, *COX1*, *CYC*, *PEPCK* and *UBC* were screened (Figure S1). GeNorm screened out the stability of the 6 candidate reference genes from high to low: *CYC&UBC >PEPCK> ACT > GAPDH > COX1* (Figure S2; Figure S3); Normfinder obtained the stability of the candidate genes from high to low was: *PEPCK > ACT > CYC > GAPDH > COX1 > UBC* (Figure S4), among which *PEPCK* was the most stable; Bestkeeper found that the stability of the 6 reference genes ranged from high to low: *CYC > UBC > PEPCK > GAPDH > ACT > COX1* (Figure S5), respectively. The two genes, *CYC* and *PEPCK* from 15 candidate housekeeping genes were determined to be suitable as reference genes for the gene expression analyses in *S. trochoidea* by using real-time fluorescence quantitative PCR.

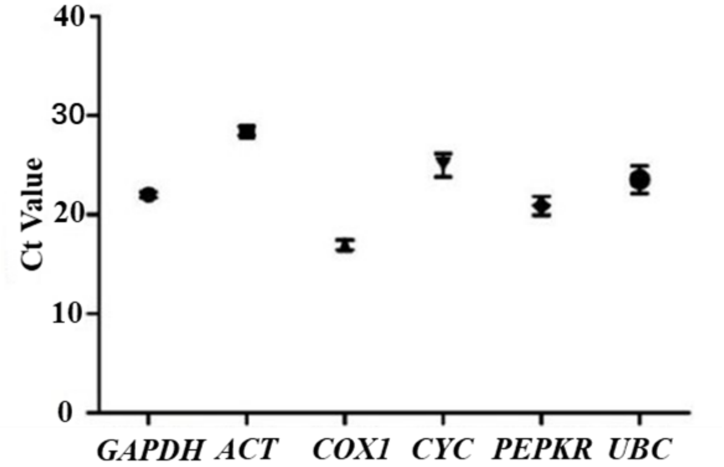

Figure S1-1 Changes of Ct values of 6 candidate housekeeping genes. The average Ct values of the 6 candidate genes are all less than 30, and the range of Ct value changes is small.

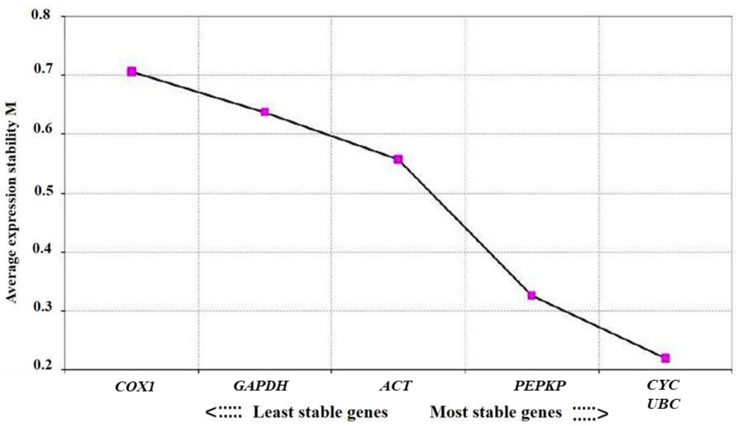

Figure S1-2 Expression stability of 6 candidate housekeeping genes calculated by GeNorm. Mean expression stability (M) removed the most unstable expressed genes gradually. Smaller M indicates more stable expression.

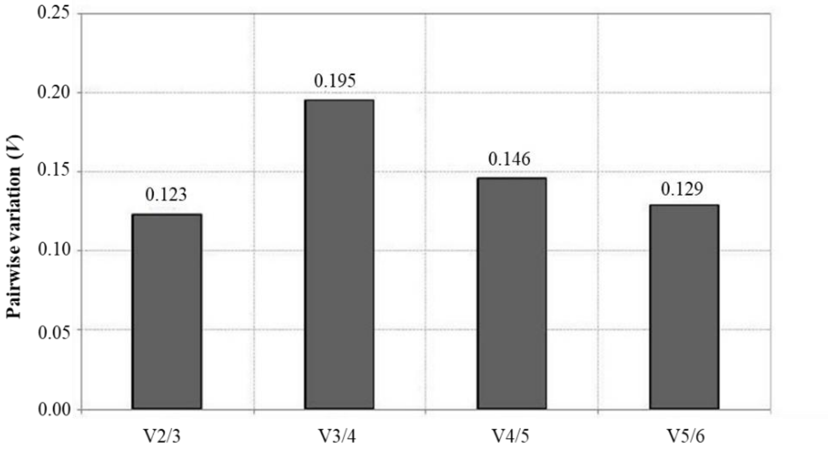

Figure S1-3 Pairwise variation of the optimal number of candidate housekeeping genes for normalization. *V* value showed the optimal internal reference gene (minimum M value) as the benchmark. And the pairwise variation ($V_{n/n+1}$) was analyzed between the normalization factor to select and determine the optimal number of internal reference genes.

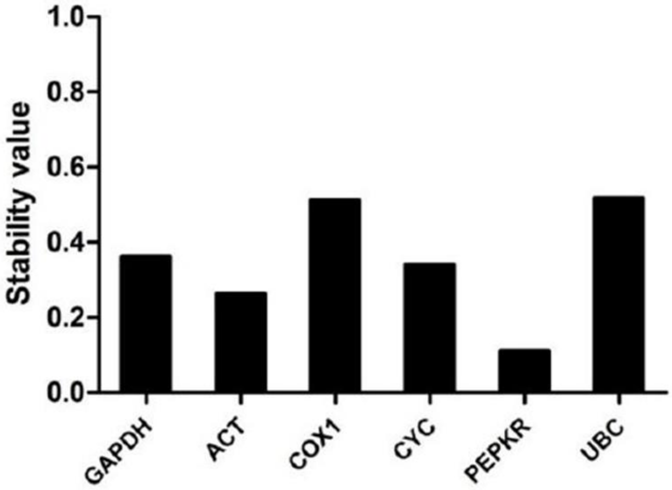

Figure S1-4 Stability values (SV) of 6 candidate housekeeping genes calculated by Normfinder. The lower the SV, the more stable the gene expression.

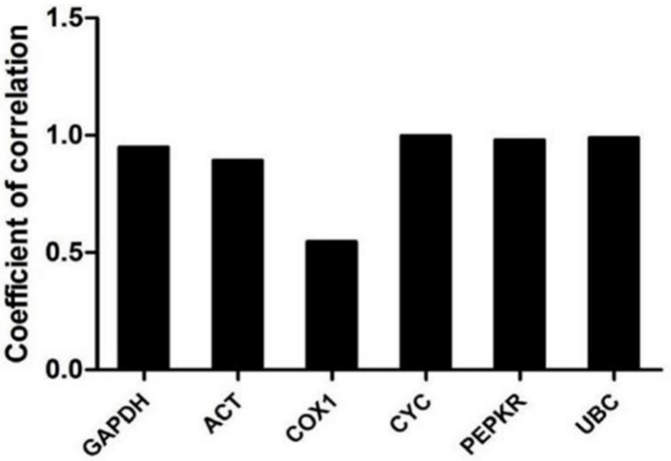

Figure S1-5 Correlation coefficient (*r*) values of 6 candidate housekeeping genes calculated by BestKeeper. The

value of standard deviation (SD) must be less than 1, which was considered as stable in expression. The *r* was the geometric mean of all stable candidate genes, and the higher *r* value indicates more stable expression pattern.

3. Conclusion

In this experiment, real-time fluorescence quantitative PCR technology and three softwares were commonly used to analyze internal reference genes. Since calculations of the 3 softwares were principally based on different algorithms, so the same gene can be calculated differently [11, 12]. *CYC* and *PEPCK* are the most suitable internal reference genes for studying the different life cycle stages of *S. trochoidea*. These genes can be used for subsequent quantitative gene expression analysis.

References

1. Guillard, R. R. Culture of phytoplankton for feeding marine invertebrates. In *Culture of marine invertebrate animals*, Springer: **1975**; pp 29-60.
2. Yang, A.; Hu, Z.; Tang, Y. Solid sand particle addition can enhance the production of resting cysts in dinoflagellates. *Journal of Oceanology and Limnology* **2018,** *36* (2), 273-280.
3. Su, J.; Yang, X.; Zheng, T.; Hong, H. An efficient method to obtain axenic cultures of *Alexandrium tamarense*—a PSP-producing dinoflagellate. *Journal of microbiological methods* **2007,** *69* (3), 425-430.
4. Deng, Y.; Hu, Z.; Shang, L.; Peng, Q.; Tang, Y. Z. Transcriptomic analyses of *Scrippsiella trochoidea* reveals processes regulating encystment and dormancy in the life cycle of a dinoflagellate, with a particular attention to the role of abscisic acid. *Frontiers in microbiology* **2017,** *8*, 2450.
5. Pfaffl, M. W. A new mathematical model for relative quantification in real-time RT–PCR. *Nucleic acids research* **2001,** *29* (9), e45-e45.
6. Radonić, A.; Thulke, S.; Mackay, I. M.; Landt, O.; Siegert, W.; Nitsche, A. Guideline to reference gene selection for quantitative real-time PCR. *Biochemical and biophysical research communications* **2004,** *313* (4), 856-862.
7. Schmittgen, T. D.; Zakrajsek, B. A.; Mills, A. G.; Gorn, V.; Singer, M. J.; Reed, M. W. Quantitative reverse transcription–polymerase chain reaction to study mRNA decay: comparison of endpoint and real-time methods. *Analytical biochemistry* **2000,** *285* (2), 194-204.
8. Vandesompele, J.; De Preter, K.; Pattyn, F.; Poppe, B.; Van Roy, N.; De Paepe, A.; Speleman, F. Accurate normalization of real-time quantitative RT-PCR data by geometric averaging of multiple internal control genes. *Genome biology* **2002,** *3* (7), 1-12.
9. Andersen, C. L.; Jensen, J. L.; Ørntoft, T. F. Normalization of real-time quantitative reverse transcription-PCR data: a model-based variance estimation approach to identify genes suited for normalization, applied to bladder and colon cancer data sets. *Cancer research* **2004,** *64* (15), 5245-5250.
10. Pfaffl, M. W.; Tichopad, A.; Prgomet, C.; Neuvians, T. P. Determination of stable housekeeping genes, differentially regulated target genes and sample integrity: BestKeeper–Excel-based tool using pair-wise correlations. *Biotechnology letters* **2004,** *26* (6), 509-515.
11. de Almeida, M. R.; Ruedell, C. M.; Ricachenevsky, F. K.; Sperotto, R. A.; Pasquali, G.; Fett-Neto, A. G. Reference gene selection for quantitative reverse transcription-polymerase chain reaction normalization during in vitro adventitious rooting in Eucalyptus globulus Labill. *BMC Molecular Biology* **2010,** *11* (1), 1-12.
12. Demidenko, N. V.; Logacheva, M. D.; Penin, A. A. Selection and validation of reference genes for quantitative real-time PCR in buckwheat (Fagopyrum esculentum) based on transcriptome sequence data.

PloS one **2011,** *6* (5), e19434.
