# Supplementary material for: Probing the Energetic Metabolism of Resting Cysts under Different Conditions from Molecular and Physiological Perspectives in the Harmful Algal Blooms-Forming Dinoflagellate Scrippsiella trochoidea
Source: Int J Mol Sci. 2021 Jul 7;22(14):7325. doi: 10.3390/ijms22147325 (PMC8307125; doi:10.3390/ijms22147325)
Supplement: Supplementary file 1 [file ijms-22-07325-s001.zip › Supplementary Materials 2.pdf]

Supplementary materials 2. Probing the energetic metabolism of resting cysts under different conditions from molecular and physiological perspectives in the harmful algal blooms-forming dinoflagellate *Scrippsiella trochoidea*

1. Characteristics analyses of *Stβ-F₁-ATPase*

Amino acid sequences of alveolata, alga, higher plants, animal and bacteria species used in our study were down-loaded from the GenBank database, including *Besnoitia besnoiti* (XP_029221878.1), *Hammondia hammondi* (KEP66196.1), *Perkinsus marinus* (XP_002782393.1), *Perkinsus olseni* (KAF4689375.1), *Neospora caninum Liverpool* (XP_003882811.1), *Cystoisospora suis* (PHJ21285.1), *Toxoplasma gondii MAS* (KFH15380.1), *Cyclospora cayetanensis* (XP_022586579.1), *Karlodinium veneficum* (ADV91188.1), *Pfiesteria piscicida* (ACU45001.1), *Saccharina japonica* (YP_006639069.1), *Ulva linza* (YP_009256600.1), *Galdieria sulphuraria* (XP_005704290.1), *Fistulifera solaris* (GAX15371.1), *Phaeodactylum tricornutum* (XP_002177917.1), *Thalassiosira pseudonana* (XP_002296041.1), *Ectocarpus siliculosus* (CBJ32298.1), *Chlamydomonas reinhardtii* (NP_958414.1), *Ostreococcus tauri* (AGW31159.1), *Cyanidioschyzon merolae* (QFV17223.1), *Emiliania huxleyi* (YP_277339.1), *Varroa destructor* (XP_022667920.1), *Cryptotermes secundus* (XP_023708910.1), *Schistocerca gregaria* (AEV89780.1), *Tropilaelaps mercedesae* (OQR69095.1), Zootermopsis nevadensis (XP_021935193.1), *Locusta migratoria* (AQE30075.1), *Homo sapiens* (AAA51808.1), *Mus musculus* (NP_058054.2), *Rattus norvegicus* (NP_599191.1), *Alphaproteobacteria bacterium* (MSO75027.1), *Rhodospirillaceae bacterium* (WP_119462901.1), *Pelagibacterales bacterium* (RZO47986.1), *Candidatus Pelagibacter* (MAH52429.1), *Hypericibacter adhaerens* (WP_151120001.1), *Azospirillum halopraeferens* (WP_029011043.1), *Abelia macrotera* (A0A5P8G1W4), *Ginkgo biloba* (Q4FGI7-1), *Arabidopsis thaliana* (NP_680155), *Agave americana* (A0A1I9QLK6-1), *Mimosa pudica* (A0A4V1GPW3- 1), *Aspidistra elatior* (Q95AF8-1), *Euryale ferox* (A0A2U3TDD9-1), *Eutrema japonicum* (A0A5H2YRI0-1), *Parthenium argentatum* (D1M7N2-1) and *Mesembryanthemum crystallinum* (A0A0G2R7U6-1). Sequences analysis was performed by DNAMAN (Figure S2-1). Identical and similar amino acid residues are black and gray shaded, respectively. Deletions are indicated by dots. In order to make the data clearer, we transfer the data to a table for presentation (Table S2-1).

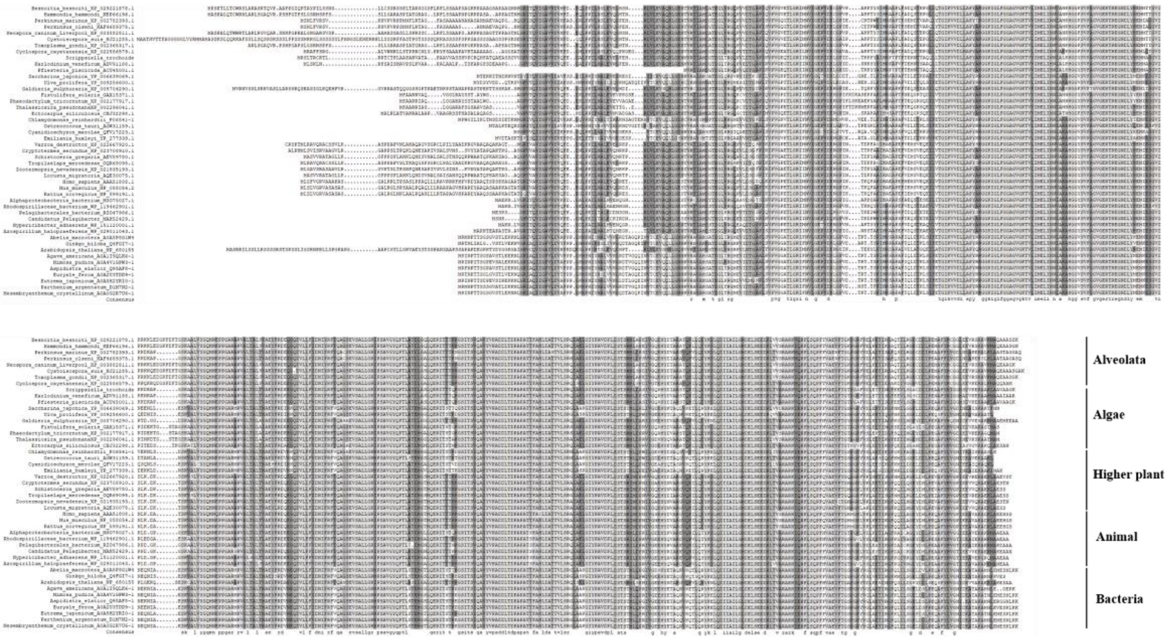

Figure S2-1. Alignment and comparison of *Stβ-F1-ATPase* deduced amino acid sequences from *Scrippsiella trochoidea* with different species. Sequence analysis was performed by DNAMAN and numbered on the left. Identical and similar amino acid residues are black and gray shaded, respectively. Deletions are indicated by dots. Details on species names and GenBank accession numbers are shown above.

Table S2-1. Amino acid sequences of alveolata, alga, higher plants, animal and bacteria species

Species	Accession number
Besnoitia besnoiti	XP_029221878.1
Hammondia hammondi	KEP66196.1
Perkinsus marinus	XP_002782393.1
Perkinsus olseni	KAF4689375.1
Neospora caninum Liverpool	XP_003882811.1
Cystoisospora suis	HJ21285.1
Toxoplasma gondii	XP_002365317.1
Cyclospora cayetanensis	XP_022586579.1
Scrippsiella trochoide	MZ343333
Karlodinium veneficum	ADV91188.1
Pfiesteria piscicida	ACU45001.1
Saccharina japonica	YP_006639069.1
Ulva prolifera	YP_009256600.1
Galdieria sulphuraria	XP_005704290.1
Fistulifera solaris	GAX15371.1
Phaeodactylum tricornutum	XP_002177917.1
Thalassiosira pseudonana	XP_002296041.1
Ectocarpus siliculosus	CBJ32298.1

Chlamydomonas reinhardtii	P06541-1
Ostreococcus tauri	AGW31159.1
Cyanidioschyzon merolae	QFV17223.1
Emiliania huxleyi	YP_277339.1
Varroa destructor	XP_022667920.1
Cryptotermes secundus	XP_023708910.1
Schistocerca gregaria	AEV89780.1
Tropilaelaps mercedesae	OQR69095.1
Zootermopsis nevadensis	XP_021935193.1
Locusta migratoria	AQE30075.1
Homo sapiens	AAA51808.1
Mus musculus	NP_058054.2
Rattus norvegicus	NP_599191.1
Alphaproteobacteria bacterium	MSO75027.1
Rhodospirillaceae bacterium	WP_119462901.1
Pelagibacterales bacterium	RZO47986.1
Candidatus Pelagibacter	MAH52429.1
Hypericibacter adhaerens	WP_151120001.1
Azospirillum halopraeferens	WP_029011043.1
Abelia macrotera	A0A5P8G1W4
Ginkgo biloba	Q4FGI7-1
Arabidopsis thaliana	NP_680155
Agave americana	A0A1I9QLK6-1
Mimosa pudica	A0A4V1GPW3-1
Aspidistra elatior	Q95AF8-1
Euryale ferox	A0A2U3TDD9-1
Eutrema japonicum	A0A5H2YRI0-1
Parthenium argentatum	D1M7N2-1
Mesembryanthemum crystallinum	A0A0G2R7U6-1
