# Supplementary material for: Probing the Energetic Metabolism of Resting Cysts under Different Conditions from Molecular and Physiological Perspectives in the Harmful Algal Blooms-Forming Dinoflagellate Scrippsiella trochoidea
Source: Int J Mol Sci. 2021 Jul 7;22(14):7325. doi: 10.3390/ijms22147325 (PMC8307125; doi:10.3390/ijms22147325)
Supplement: Supplementary file 1 [file ijms-22-07325-s001.zip › Supplementary Materials 3.pdf]

Supplementary materials 3. Probing the energetic metabolism of resting cysts under different conditions from molecular and physiological perspectives in the harmful algal blooms-forming dinoflagellate *Scrippsiella trochoidea*

Table S3-1. Information of nutrient concentrations (Guillard, 1975)

**Component**	**Molar Concentration in Final Medium**
$NaNO_3$	8.82×10^{-4} M
$NaH_2PO_4 \cdot H_2O$	3.62×10^{-5} M
$FeCl_3 \cdot 6H_2O$	1.17×10^{-5} M
$Na_2EDTA \cdot 2H_2O$	1.17 × 10-5 M
CuSO4 $5H_2O$	3.93×10^{-8} M
$Na_2MoO_4 \cdot 2H_2O$	2.60×10^{-8} M
$ZnSO_4 \cdot 7H_2O$	7.65×10^{-8} M
$CoCl_2 \cdot 6H_2O$	4.20×10^{-8} M
$MnCl_2 \cdot 4H_2O$	9.10×10^{-7} M
thiamine HCl (vit. B_1)	2.96×10^{-7} M
biotin (vit. H)	2.05×10^{-9} M
cyanocobalamin (vit. B_{12})	3.69×10^{-10} M

Reference:

Guillard, R. R. Culture of phytoplankton for feeding marine invertebrates. *Culture of marine invertebrate animals* **1975**, pp 29-60.
